# Whole genome sequencing in animal health: applications, challenges, and future directions

**DOI:** 10.1186/s12917-026-05329-7

**Published:** 2026-02-06

**Authors:** Johannes Charlier, Alistair Antonopoulos, Bart J.G. Broeckx, Aaron Pomerantz, Veronica L Fowler, Volodimir Olexiouk, Sebastiaan Theuns, Sven Arnouts

**Affiliations:** 1 Kreavet, Hendrik Mertensstraat 17, Kruibeke, 9150 Belgium; 2 Laboratory of Animal Genetics, Department of Veterinary and Biosciences, Faculty of Veterinary Medicine, Ghent University, Merelbeke, Belgium; 3 Oxford Nanopore Technologies Plc, Gosling Building, Edmund Halley Road, Oxford Science Park, Oxford, OX44DQ UK; 4 BioLizard NV, Foreestelaan 88 - 2nd floor, Ghent, 9000 Belgium; 5 PathoSense BV, Poortakkerstraat 41A, Ghent, 9051 Belgium; 6 Provaxs, Department of Translational Physiology, Infectiology and Public Health, Ghent University, Merelbeke, Belgium

**Keywords:** Whole genome sequencing, Animal health, Veterinary, Diagnostics, Molecular diagnostics

## Abstract

WGS is revolutionizing animal health diagnostics, offering unprecedented opportunities for pathogen detection and biomarker identification, while offering increased depth of data, in comparison to more targeted approaches such as amplicon sequencing. However current diagnostic approaches stay valuable as they are often still more cost-effective and easier to interpret. WGS can, however, offer the ability to carry out species identification and combine this with additional insights such as presence of antimicrobial resistance (AMR) mutations or genes, vaccine escape variants, recombination or reassortment, and virulence and pathogenicity factors. Moreover, the technology enables more systematic approaches, capable of screening the whole pathogenome or host genome instead of the traditional diagnostic approaches where a selection of pathogens or diagnostic markers needs to be made beforehand with a high chance of missing the (interplay of) causative agent(s) that has led to the disease. While already transforming epidemiological surveillance, practical diagnostic implementation remains challenging in some contexts due to genome complexity, sample processing requirements and accessibility of the technology. We make some recommendations on how research can overcome the remaining challenges to unlock the full potential of WGS technologies in the diagnosis of animal diseases.

## Introduction: accelerating delivery of whole genome sequencing at a global level

Whole genome sequencing (WGS) has emerged as a novel tool in human and animal health, offering unprecedented insights into the genetic underpinnings of diseases and transforming current diagnostic approaches. Here, we define WGS in the broadest sense, as any sequencing approach that seeks to sequence the entire genome of the pathogen in question, whether it is a viral genome of ~ 10 kb, or a eukaryotic microparasite genome > 2.8 Mb. In this sense, we set out to discuss how targeting the entire genome of the pathogen can improve animal health moving forward. This can be via improved epidemiological surveillance, detection of unknown pathogens, or the elucidation of novel resistance markers. WGS has become a powerful means to integrate several new applications into the routine of veterinary laboratories: from accurate characterisation of pathogens to screening for presence of AMR mutations or genes, vaccine escape variants, recombination or reassortment, and virulence and pathogenicity factors [1]. This technology enables more systematic approaches, capable of screening the whole pathogenome or host genome instead of the traditional diagnostic approaches where a selection of pathogens or diagnostic markers needs to be made beforehand with a high chance of missing the (interplay of) causative agent(s) that has led to the disease.

Despite its remarkable potential, WGS also faces challenges limiting its widespread implementation in veterinary diagnostics. These included the need for standardized and validated bioinformatic pipelines and reference databases and clear data interpretation guidelines suitable for different animal species and pathogens [2]. Moreover, high computational demands, access to reagents and trained staff can limit its uptake, particularly in resource limited settings.

This narrative review aims to summarise current applications, challenges, and future directions for WGS in animal health. The review is informed by discussions held during the Animal Health Nutrition Technology Innovation Europe workshop (London, February 2025), organised by Provaxs – UGent and STAR IDAZ IRC. In this review we aim to discuss (i) where the technology stands at the moment and how it will further evolve; (ii) how bioinformatics fueled by artificial intelligence is unlocking diagnostic information that was previously inaccessible; (iii) which cost-effective diagnostic approaches are already possible today; (iv) which major research gaps could be filled by deployment and further advancement of the technology and finally (v) which risks and threats should be considered for the fair and equitable use and accessibility of the technology around the globe.

### WGS made cost-effective and accessible to the animal health sector

Advances in sequencing technologies are reshaping the landscape of veterinary diagnostics, research, and disease surveillance. WGS, once limited to well-funded research institutions, is now increasingly accessible to veterinary laboratories, field settings, and public health agencies, with WGS now mandatory for specific foodborne pathogens in all EU member states [3]. The ability to obtain complete genomic information on pathogens, microbiomes, or host species in a single assay would, in some cases, particularly when dealing with small viral genomes, potentially allow for more accurate and timely responses to disease outbreaks and zoonotic threats. In animal health, this translates into more precise epidemiological tracing, and informed treatment or control strategies across livestock, companion animals, and wildlife populations [4].

A key driver of this accessibility has been the emergence of portable, real-time, and cost-effective sequencing platforms that eliminate the need for centralized infrastructure or batching requirements [5]. Among these, technologies such as those offered by Oxford Nanopore Technologies are helping veterinary and public health professionals analyze atypical or mixed infections in a hypothesis-free manner, detect AMR markers, and resolve entire microbial genomes and plasmids from diverse species [6]. These tools are already being implemented for rapid surveillance of economically important pathogens such as Salmonella [7], African swine fever virus [8, 9], Foot-and-mouth disease virus [10–12] and Avian influenza [13–15], as well as for microbiome or genetic diseases studies that inform animal health, welfare, and productivity. With that in mind, however, it must still be stated that existing platforms such as Illumina and PacBio still offer significant advantages, and when sequencing facilities are available and accessible, may still remain the preferred platform.

Importantly, simplified sample preparation protocols and cloud-based automated analysis options have lowered technical barriers, enabling smaller labs or field units to carry out comprehensive genomic investigations [7–15]. As sequencing becomes more integral to biosecurity, animal breeding, vaccine design, and antimicrobial stewardship, the democratization of WGS represents a turning point for veterinary medicine. The future of animal health will rely not only on innovation but on widespread, affordable access to genomic tools.

### Revolutionary and complete diagnostics of infectious diseases

The ongoing development of high-throughput sequencing technologies has fundamentally revolutionized the way biological and evolutionary processes can be studied at the molecular level. This new technology comes timely in the turning point where increasing globalization, agricultural intensification, urbanization, and climatic changes are significantly increasing emerging infectious transboundary and zoonotic disease risks. It enables innovative, effective, and integrative research, which is essential to better understand infectious disease transmission, ecological implications, and dynamics at wildlife-human interfaces [16].

Whole-genome sequencing methodologies have enormous potential for unravelling these contingencies and improving our understanding of pathogen detection and surveillance. The second and third-generation sequencing platforms provide numerous benefits over traditional microbiological diagnostic techniques, including the ability to detect fastidious or non-culturable pathogens and co-infections. The latter is important as many livestock and companion animal diseases are caused by complexes in which multiple pathogens can contribute. Traditional diagnostics also focus on known pathogens, while sequencing-based approaches allow the discovery of novel pathogens and the detection of neglected viruses and bacteria. Sequencing technologies enable researchers to simultaneously identify a wide range of DNA and RNA sequences, either using a specific genetic region (metabarcoding/amplicon-based methods) or by random reading of all genetic material (metagenomics), leading to unprecedented throughput and more accurate responses to disease outbreaks, antimicrobial resistance, and zoonotic threats. The technology also has the potential to deliver quicker responses to outbreaks, for instance when new (viral) strains are implied that are not included in regular, targeted surveillance programmes. This has already been proven in human medicine [17], in addition to the discovery of the Schmallenberg virus in 2011, which was first identified as a novel orthobunyavirus in cattle using a metagenomic approach [18]. Although metabarcoding and amplicon sequencing technologies are technically separate from WGS based techniques, the two exist interdependently and synergistically, and amplicon-based technologies are particularly dependent on good quality reference genomes for accurate primer design, and novel target gene identification [7]. To address diverse diagnostic issues, companies are developing relatively affordable, rapid, and random sequencing approaches for the rapid detection of infections in animals without prior selection of putative pathogens. This makes WGS technologies already available for clinical veterinarians [19–22]. This represents a move toward the inclusion of newer technologies to improve understanding of disease dynamics using comprehensive genomic approaches, rather than reliance solely on traditional culture-based, or amplification-based methods [16]. Current challenges can be found in the scaling of this technology to all animal species, requiring species-specific interpretation of disease complexes. In the future, such WGS diagnostic approaches may also include virulence factor screening, AMR detection, and an in-depth understanding of the microbiome present in healthy versus diseased hosts, which can theoretically be integrated into a single analysis pipeline.

### Animal genomics for accurate diagnosis and treatment of genetic diseases

Companion animals, like cats and dogs in particular, do not only suffer from genetic diseases similar or identical to humans, in some cases they encounter these genetic diseases at frequencies that are many times higher [23]. Public awareness of these issues has led to breeding legislation in several countries [24]. Legal measures do not resolve everything however, and it remains key to act on risk reduction and provide tailored breeding advice [23, 25]. Just as in humans, it is important to realize that genetic disease will always keep on occurring, which substantiates the need for genetic counselling and clinical genetics [24]. The need for fast and accurate diagnostic tools for (companion) animal genetic diseases is clear and WGS and whole exome sequencing are making this now possible. These tools are not only suitable for screening purposes [26], but can also be used for the identification of new disease-causing variants [27] and will in the near future likely become an integral part of the toolbox, next to more traditional techniques like Sanger sequencing, TaqMan probes and so on. The need extends beyond so-called simple Mendelian diseases. Especially in cancer, the word “fast” is critical as some forms of cancer are (extremely) aggressive and associated with short survival times [28]. Delays linked to the diagnostical work-up should thus be limited as much as possible and this is where fast genomic and computational approaches can make the difference, also by allowing a more tailored treatment [29, 30]. Same-day results or even within minutes have become realistic thanks to the real-time analysis of Oxford Nanopore Technologies data, combined with fast artificial intelligence-based algorithms. None of this however is without challenges: it requires bioinformatics tools and analytic frameworks to interpret al.l this data [31, 32] and steps are to be taken to develop this further. While the knowledge to develop this is certainly present in veterinary medicine, the relatively limited existence of biobanks and funding limitations are named as the aspects that hinder fast implementation.

### Bioinformatics turning complex data into clear diagnostic results

Modern bioinformatics pipelines for WGS have evolved into robust, user-friendly workflows that streamline data analysis. Community-curated pipelines, such as those from the nf-core project (https://nf-co.re/), now offer standardized, end-to-end analyses that relieve researchers from developing custom code for every study [33]. This commoditization shifts the emphasis toward optimizing parameters, interpreting biological relevance, and rigorously validating results. Interactive platforms further democratize these analyses by enabling scientists and clinicians to execute complex workflows via intuitive interfaces and share reproducible results [34]. However, as important as these developments are, there are still multiple steps involving sampling and sample preparation that may still affect the outcome of the sequencing run [35–37].

It is important to recognize that all diagnostic and bioinformatic methods serve as proxies for underlying biological reality. Every output, whether a list of genetic variants, inferred pathogen lineages, or predicted drug resistance profile, is derived from indirect measurements and inherent assumptions. Biases arise when limited or unrepresentative datasets are used. For example, reliance on a single reference genome may overlook the true genetic diversity within animal populations [38]. This issue is particularly acute in veterinary genomics, where many breeds or subpopulations remain underrepresented, leading to systematic under-detection of breed-specific variants and other clinically relevant variables [38, 39]. Therefore, computational results should be regarded as hypotheses that require experimental validation. To mediate this, integrating wet-lab and dry-lab workflows is essential for converting WGS data into reliable diagnostics. Dry-lab analyses generate hypotheses, such as flagging a novel mutation as a candidate disease driver, that can be tested experimentally. This iterative feedback loop refines both diagnostic tools and computational models [40].

WGS, and diagnostics in general, also benefit tremendously from advances achieved in human genomics. Technologically, the value of WGS has matured as costs decline and data quality improves. Analytically, biological insights derived from model organisms and human studies can be translated into the veterinary domain to fill knowledge gaps and accelerate research. Just as drugs used in human medicine are often repurposed for animal care, information from human and model-organism studies can be leveraged to uncover new diagnostic and therapeutic opportunities in animals. A notable example is a physiological translator, which employs graph convolutional networks to integrate and map molecular data from distinct species while preserving species-specific patterns [41, 42]. This approach not only accelerates biomarker discovery but also informs targeted interventions, thereby broadening the scope and impact of WGS based diagnostics in animal health. By coupling standardized bioinformatics pipelines with iterative wet-lab validation and drawing on translational insights from human genomics, researchers are now better equipped to interpret complex genomic data and drive impactful clinical decisions [43].

### Research needs to fuel the widespread adoption of WGS in animal health

STAR IDAZ IRC contributes to the global animal health research agenda through the organisation of workshops, developing research roadmaps and recommendations for future research. STAR IDAZ developed a generic roadmap for diagnostics in animal health [44]. The roadmap starts with research on sample type or preparation up to a fit-for-purpose diagnostic tool that would be ready for technology transfer to a commercial application. These roadmaps have been developed for major livestock diseases, like Foot and Mouth Disease (FMD), bovine Tuberculosis (bTB) and helminths. So, we can ask ourselves which remaining diagnostic bottlenecks could be resolved via WGS?

When considering FMD, WGS is considered a lever to detect multiple circulating variants and serotypes in a single sample, to better understand the virus evolution and selection within the host and to validate and improve field diagnostics [44, 45]. When considering bTB, WGS holds a lot of potential to identify new biomarkers to improve the accuracy of the current diagnostic tests and to differentiate/classify bTB strains and assess their zoonotic or pandemic potential. Moreover, WGS driven research could finally make us understand why bTB benefits from major antigens being highly conserved and under purifying selection [46, 47]. While the applications of WGS in macroparasites have been slower than in viruses or bacteria, because their genomes are larger and more complex, also here WGS is now rapidly unlocking new possibilities. In helminth and anthelmintic resistance research, for instance, WGS has the potential to replace the widely used faecal egg counting technique offering multiple additional diagnostic information at similar cost. Assessing the full parasite species composition in a sample while, at the same time, detecting antiparasitic resistance mutations has the potential to crack the code of antiparasitic resistance markers and offer user friendly antiparasitic resistance test in the field, guiding smarter treatment decisions [7, 48]. STAR IDAZ experts also call for the development of a standardised nomenclature linking the old molecular techniques like Multiple Locus Variable-number Tandem Repeat Analysis and spoligotyping of *Mycobacterium bovis* with the WGS data. Finally, a recent diagnostic gap analysis showed that several high priority diseases in animal health still lack the wide availability of commercial diagnostic tests, in particular for zoonotic diseases [49]. A question to be answered through further research is if WGS can fill this diagnostic gap by the development of cost-effective and accessible ‘complete’ diagnostic approaches. Finally, a key area where further research is necessary and will be beneficial is the exploration of the use of adaptive sampling for WGS using nanopore sequencing. Adaptive sampling, where target sequences are actively enriched during sequencing by live alignment to reference sequences, and are then discarded if there is an insufficient match, has been shown to greatly improve the efficiency of detection of microbial reads in samples dominated by host DNA [50]. However, there is still further work necessary to apply this to all pathogens and sample types, with mixed results when using adapative sampling for WGS applications in helminth samples [51]. A summary of the different sequencing methodologies, and their advantages and limitations can be found in Table 1.

**Table 1:** Summary of advantages and limitations of different sequencing methodologies for animal health

Sequencing Method	Advantages	Limitations
Metagenomics	Allows for study of all genetic material, complex interactions (i.e. microbiome), culture independent, direct analysis	Background noise in the sample, low frequency species/variants can be missed, computationally demanding to process data
Targeted (amplicon) sequencing/Metabarcoding	Sequence only the gene(s) or region(s) of interest, allows for amplification of region(s) of interest reducing background noise	Amplification bias, can miss low frequency variants, requires prior knowledge of region(s) of interest
WGS	Captures the whole genome of the pathogen(s) of interest, allows for future reanalysis of historic samples without need for resequencing, valuable for identifying resistance/virulence mutations, novel stain/species detection	Complex to process samples, labour intensive sample preparation, computationally demanding for genome assembly (particularly for large genomes)

### Opportunities or threats?

The discussion that followed the introductory presentations on the technology (WGS and bioinformatics) and examples of its applications in animal health reflected a wide range of perspectives on the transformative potential of WGS, as well as the challenges associated with its widespread adoption.

Supporters of NGS emphasized its unparalleled speed, accuracy, and ability to detect a broad spectrum of pathogens and genetic markers, positioning it as a valid or superior alternative to traditional diagnostics like culture-based methods, PCR, and serology. WGS using ever improving and more cost effective NGS technologies offers the advantage of increased density of data, when compared to more targeted approaches. With decreasing costs and advancements in real-time sequencing, the use of WGS in veterinary diagnostics is expected to increase, enabling earlier detection, precision medicine, and targeted interventions. Its potential to detect asymptomatic carriers and genetic predispositions could help prevent disease outbreaks and reduce long-term healthcare costs. This is particularly important when considering pathogens such as retroviruses, which can lie dormant for long periods of time integrated into the host genome, such as the case of HIV in humans [52]. In the veterinary field, small ruminant lentiviruses also lie latent in the host body for several years, prior to causing active infection and disease [53]. Therefore, in future WGS could serve as an early detection method for such diseases. Within the field of resistance marker identification, WGS has played a key role in elucidating benzimidazole, macrocyclic lactone, and levamisole markers of gastroeintestinal helminths, and fluke [54, 55]. WGS plays an essential role in linking of phenotype (i.e. clinically resistant isolates) with genotype (genetic markers/mechanisms of resistance), by comparative genomic analysis of resistant and susceptible populations prior to, and post, drug treatment [54, 55].

However, concerns were raised about the accessibility and affordability of WGS, particularly in resource-limited settings where traditional methods remain practical and cost-effective. There are also concerns on the possibility of over-diagnosis, where the detection of minor mutations or latent infections could lead to unnecessary treatments, excessive medical interventions, and even unjustified culling in livestock industries. Moreover, detection of mutations during screening can cause unnecessary concern to owners and the ethics of such screening need to be considered carefully. The discussion underscored the importance of clinical diagnosis, integrated with high-quality sequencing data and well-validated bioinformatics pipelines, to prevent wrong diagnoses and unnecessary responses following detection of putative pathogens in otherwise healthy subjects.

## Conclusions

While WGS will play an increasingly important role in animal healthcare, it is unlikely to completely replace conventional diagnostics. Instead, a balanced integration of WGS with traditional methods, supported by rigorous validation and ethical considerations, will be key to maximizing its benefits while minimizing risks. A number of key challenges remain, particularly, but not limited to, current availability of sequencing facilities, the computational power and expertise needed to accurately analyse WGS data, and the size of some pathogen genomes making WGS impractical at the time of writing. However, technology continues to improve, and newer portable sequencing technologies such as nanopore are now beginning to open the pathway to the wider implementation of sequencing across the veterinary field.

## Data Availability

Not applicable.

## Abbreviations

AMR: Antimicrobial resistance
bTB: Bovine tuberculosis
DNA: Deoxyribonucleic acid
FMD: Foot and mouth disease
PCR: Polymerase chain reaction
STAR IDAZ IRC: Global Strategic Alliances for the Coordination of Research on the Major Infectious Diseases of Animals and Zoonoses - International Research Consortium
WGS: Whole genome sequencing

## References

[1] Charlier J, Barkema HW, Becher P, De Benedictis P, Hansson I, Hennig-Pauka I, La Ragione R, Larsen LE, Madoroba E, Maes D, Marín CM, Mutinelli F, Nisbet AJ, Podgórska K, Vercruysse J, Vitale F, Williams DJL, Zadoks RN. Disease control tools to secure animal and public health in a densely populated world. Lancet Planet Health. 2022;6(10):e812–24. 36208644 10.1016/S2542-5196(22)00147-4

[2] Doyle SR. Improving helminth genome resources in the post-genomic era. Trends Parasitol. 2022;38:831–40. 10.1016/j.pt.2022.06.002. 35810065 10.1016/j.pt.2022.06.002

[3] Gomes E, Araújo D, Nogueira T, Oliveira R, Silva S, Oliveira LVN, Azevedo NF, Almeida C, Castro J. Advances in whole genome sequencing for foodborne pathogens: implications for clinical infectious disease surveillance and public health. Front Cell Infect Microbiol. 2025;15:1593219. 10.3389/fcimb.2025.1593219. 40357405 10.3389/fcimb.2025.1593219 PMC12066639

[4] Ji CM, et al. The applications of nanopore sequencing technology in animal and human virus research. Viruses. 2024;16(5):798. 38793679 10.3390/v16050798 PMC11125791

[5] Kafetzopoulou LE, et al. Metagenomic sequencing at the epicenter of the Nigeria 2018 Lassa fever outbreak. Science. 2019;363(6422):74–7. 30606844 10.1126/science.aau9343 PMC6855379

[6] Elton L et al. A metagenomic approach to One Health surveillance of antimicrobial resistance in a UK veterinary centre. bioRxiv, 2025: 2025-02. 10.1099/mgen.0.001471 PMC12401494 40889140

[7] Wu X, et al. Evaluation of multiplex nanopore sequencing for Salmonella serotype prediction and antimicrobial resistance gene and virulence gene detection. Front Microbiol. 2023;13:1073057. 36817104 10.3389/fmicb.2022.1073057 PMC9930645

[8] Warr A, Newman C, Craig N, Vendele I, Pilare R, Cariazo Cruz L, Galase Barangan T, Morales RG, Opriessnig T, Venturina M, Mananggit V, Lycett MR, Domingo S, Tait-Burkard CYJ. C. No part gets left behind: Tiled nanopore sequencing of whole ASFV genomes stitched together using Lilo: Tiled amplicon sequencing with improved assembly of African Swine Fever Virus. bioRxiv, 2021:1–35. 10.1101/2021.12.01.470769.

[9] Goatley LC, Freimanis G, Tennakoon C, Foster TJ, Quershi M, Dixon LK, et al. Full genome sequence analysis of African swine fever virus isolates from Cameroon. PLoS ONE. 2024;19(3):e0293049. 10.1111/journal.pone.0293049. 38512923 10.1371/journal.pone.0293049 PMC10956809

[10] Brown E, Freimanis G, Shaw AE, Horton DL, Gubbins S, King D. Characterising Foot-and-Mouth disease virus in clinical samples using nanopore sequencing. Front Veterinary Sci. 2021;8:656256. 10.3389/fvets.2021.656256. 10.3389/fvets.2021.656256 PMC8165188 34079833

[11] Hansen S, Dill V, Shalaby MA, Eschbaumer M, Böhlken-Fascher S, Hoffmann B, Czerny CP, Wahed AE, A. Serotyping of foot-and-mouth disease virus using Oxford nanopore sequencing. J Virol Methods. 2019;263:50–3. 10.1016/j.jviromet.2018.10.020. 30393148 10.1016/j.jviromet.2018.10.020

[12] Sun S, Guo H, Yin S. Analysis of coinfection pathogens from Foot-and-Mouth disease virus persistently infected cattle using Oxford nanopore sequencing. Transbound Emerg Dis. 2024;2024(9703014). 10.1155/2024/9703014. 10.1155/2024/9703014 PMC12016752 40303157

[13] Ratcliff JD, Merritt B, Gooden H, Siegers JY, Srikanth A, Yann S, Kol S, Sin S, Tok S, Karlsson EA, Thielen PM. Improved resolution of avian influenza virus using Oxford nanopore R10 sequencing chemistry. Microbiol Spectr. 2024;12:e01880–24. 10.1128/spectrum.01880-24. 39508569 10.1128/spectrum.01880-24 PMC11623064

[14] Perlas A, Reska T, Croville G, Tarrés-Freixas F, Guérin JL, Majó N, Urban L. Improvements in RNA and DNA nanopore sequencing allow for rapid genetic characterization of avian influenza. Virus Evol. 2025;11(1):veaf010. 10.1093/ve/veaf010. 40066328 10.1093/ve/veaf010 PMC11892550

[15] Croville G, Walch M, Sécula A, Lèbre L, Silva S, Filaire F, Guérin JL. An amplicon-based nanopore sequencing workflow for rapid tracking of avian influenza outbreaks, France, 2020–2022. Front Cell Infect Microbiol. 2024;14:1257586. 10.3389/fcimb.2024.1257586. 38318163 10.3389/fcimb.2024.1257586 PMC10839014

[16] Suminda GGD, Bhandari S, Won Y, Goutam U, Pulicherla KK, Son Y-O, Ghosh M. High-throughput sequencing technologies in the detection of livestock pathogens, diagnosis, and zoonotic surveillance. Comput Struct Biotechnol J. 2022;20:5378–92. 36212529 10.1016/j.csbj.2022.09.028 PMC9526013

[17] Jackson BR, Tarr C, Strain E, Jackson KA, Conrad A, Carleton H, Katz LS, Stroika S, Gould LH, Mody RK, Silk BJ, Beal J, Chen Y, Timme R, Doyle M, Fields A, Wise M, Tillman G, Defibaugh-Chavez S, Kucerova Z, Sabol A, Roache K, Trees E, Simmons M, Wasilenko J, Kubota K, Pouseele H, Klimke W, Besser J, Brown E, Allard M, Gerner-Smidt P. Implementation of nationwide real-time whole-genome sequencing to enhance listeriosis outbreak detection and investigation. Clin Infect Dis. 2016;63(3):380–6. 27090985 10.1093/cid/ciw242 PMC4946012

[18] Hoffmann B, Scheuch M, Höper D, Jungblut R, Holsteg M, Schirrmeier H, Eschbaumer M, Goller KV, Wernike K, Fischer M, Breithaupt A, Mettenleiter TC, Beer M. Novel orthobunyavirus in Cattle, Europe, 2011. Emerg Infect Dis. 2012;18(3):469 – 72. 10.3201/eid1803.111905. PMID: 22376991. 10.3201/eid1803.111905 PMC3309600 22376991

[19] Antonopoulos A, Gilleard JS, Charlier J. Next-generation sequencing technologies for helminth diagnostics and surveillance in ruminants: shifting diagnostic barriers. Trends Parasitol. 2024;40(6):511–26. 38760257 10.1016/j.pt.2024.04.013

[20] Bokma J, Vereecke N, Pas ML, Chantillon L, Vahl M, Weesendorp E, Deurenberg RH, Nauwynck H, Haesebrouck F, Theuns S, Boyen F, Pardon B. Evaluation of nanopore sequencing as a diagnostic tool for the rapid identification of Mycoplasma Bovis from individual and pooled respiratory tract samples. J Clin Microbiol. 2021;59(12):e0111021. 10.1128/JCM.01110-21. 34550807 10.1128/JCM.01110-21 PMC8601226

[21] VereeckeN, Zwickl S, Gumbert S, Graaf A, Harder T, Ritzmann M, Lillie-Jaschniski K,Theuns S, Stadler J. Viral and Bacterial Profiles in Endemic Influenza A VirusInfected Swine Herds Using Nanopore Metagenomic Sequencing on TracheobronchialSwabs. Microbiol Spectr. 2023 Feb 28;11(2):e0009823 10.1128/spectrum.00098-23 PMC10100764 36853049

[22] Malik M, Chiers K, Theuns S, Vereecke N, Chantziaras I, Croubels S, Maes D. Porcine ear necrosis: characterization of lesions and associated pathogens. Vet Res. 2023;54(1):85. 10.1186/s13567-023-01218-1 PMC10543831 37773143

[23] Broeckx B. The dog 2.0 : lessons learned from the past. Theriogenology. 2020;150:20–6. 10.1016/j.theriogenology.2020.01.043. 32000992 10.1016/j.theriogenology.2020.01.043

[24] Broeckx B. Incorporating genetic testing into a breeding program. Veterinary Clin North America-Small Anim Pract. 2023;53(5):951–63. 10.1016/j.cvsm.2023.04.002. 10.1016/j.cvsm.2023.04.002 37221103

[25] Adant L, Szymczak V, Bhatti S, Smets P, Saunders J, Peelman L, Broeckx B. Genetic counseling in veterinary medicine: towards an evidence-based definition for the small animal practice. BMC Vet Res. 2025;21(1). 10.1186/s12917-025-04495-4. 10.1186/s12917-025-04495-4 PMC11846242 39987142

[26] Boeykens, F., Hermans, M., Adant, L., De Jonge, Deboutte, B., Vandekerckhove, L.,Stock, E., Kromhout, K., Morin, A., Saunders, J., … Broeckx, B. (2024). The utilization of the Vezzoni modified Badertscher distension device in breeding programs: heritability estimates and effect on the hip dysplasia prevalence. PLoS One, 19(8). 10.1371/journal.pone.0308984. 10.1371/journal.pone.0308984 PMC11335157 39163383

[27] Boeykens F, Bogaerts E, Vossaert L, Peelman L, Van Nieuwerburgh F, Saunders J, Broeckx B. Whole exome sequencing as a screening tool in dogs: A pilot study. Comput Struct Biotechnol J. 2025a;27:960–8. 10.1016/j.csbj.2025.03.008. 40151526 10.1016/j.csbj.2025.03.008 PMC11946360

[28] Boeykens F, Hermans M, Adant L, De Jonge B, Chiers K, Bossens K, Broeckx B. A frameshift variant in the SLC6A5 gene is associated with startle disease in a family of old english sheepdogs. Anim Genet. 2025b;56:e70003. 10.1111/age.70003. 40012122 10.1111/age.70003

[29] Valli,V. E., Kass, P. H., Myint, M. S. & Scott, F. (2013). Canine Lymphomas:Association of Classification Type, Disease Stage, Tumor Subtype, Mitotic Rate,and Treatment With Survival. Veterinary Pathology, 50, 738–748 10.1177/0300985813478210 23444036

[30] Kuschel LP, Hench J, Frank S et al. Robust methylation-based classification of brain tumours using nanopore sequencing. Neuropathol Appl Neurobiol. 2023; 49(1):e12856. 10.1111/nan.12856 36269599

[31] Boeykens F, Bhatti S, Peelman L, Broeckx B. VariantscanR : an R-package as a clinical tool for variant filtering of known phenotype-associated variants in domestic animals. BMC Bioinformatics. 2023;24(1). 10.1186/s12859-023-05426-6. 10.1186/s12859-023-05426-6 PMC10394849 37528412

[32] Boeykens, F., Abitbol, M., Anderson, H., Casselman, I., de Citres, C. D., Hayward, J. J., … Broeckx, B. Development and validation of animal variant classification guidelines to objectively evaluate genetic variant pathogenicity in domestic animals. Frontiers in Veterinary Science, 2024; 11. 10.3389/fvets.2024.1497817 10.3389/fvets.2024.1497817 PMC11656590 39703406

[33] Faulhaber MM, Tardy F, Gautier-Bouchardon AV, Öfner S, Theuns S, Coppens S, Müller E, Pees M, Marschang RE. Identifying infectious agents in snakes (Boidae and Pythonidae) with and without respiratory disease. Anim (Basel). 2025;15(15):2187. 10.3390/ani15152187. 10.3390/ani15152187 PMC12345485 40804977

[34] Ewels PA et al. The nf-core framework for community-curated bioinformatics pipelines. Nature Biotechnology, 2020;38: 276–278. 10.1038/s41587-020-0439-x. Galaxy Community. (2022). 10.1038/s41587-020-0439-x 32055031

[35] Spatz S, Afonso CL. Non-Targeted RNA sequencing: towards the development of universal clinical diagnosis methods for human and veterinary infectious diseases. Veterinary Sci. 2024;11(6):239. 10.3390/vetsci11060239. 10.3390/vetsci11060239 PMC11209166 38921986

[36] Isaic A, Motofelea N, Hoinoiu T, Motofelea AC, Leancu IC, Stan E, Gheorghe SR, Dutu AG, Crintea A. Next-Generation sequencing: A review of its transformative impact on cancer Diagnosis, Treatment, and resistance management. Diagnostics (Basel). 2025;15(19):2425. 10.3390/diagnostics15192425. 41095644 10.3390/diagnostics15192425 PMC12523276

[37] Elbehiry A, Abalkhail A. Metagenomic Next-Generation sequencing in infectious diseases: clinical Applications, translational Challenges, and future directions. Diagnostics. 2025;15(16):1991. 10.3390/diagnostics15161991. 40870843 10.3390/diagnostics15161991 PMC12384723

[38] The Galaxy platform. For accessible, reproducible and collaborative biomedical analyses: 2022 update. Nucleic Acids Res, 50, W345–51. 10.1093/nar/gkac292 10.1093/nar/gkac247 PMC9252830 35446428

[39] Sherman RM, Salzberg SL. Pan-genomics in the human genome era. Nat Rev Genet. 2020;21:243–54. 10.1038/s41576-020-0212-2. 32034321 10.1038/s41576-020-0210-7 PMC7752153

[40] Morrill KE, et al. Ancestry-inclusive dog genomics challenges popular breed stereotypes. Science. 2022;376:eabk0639. 10.1126/science.abk0639. 35482869 10.1126/science.abk0639 PMC9675396

[41] van Jansen MJ, et al. Exploiting bacterial whole-genome sequencing data for evaluation of diagnostic assays: Campylobacter species identification as a case study. J Clin Microbiol. 2016;54:2882–90. 10.1128/JCM.01245-16. 27733632 10.1128/JCM.01522-16 PMC5121375

[42] Wang T, et al. MOGONET integrates multi-omics data using graph convolutional networks allowing patient classification and biomarker identification. Nat Commun. 2021;12:3445. 10.1038/s41467-021-23774-w. 34103512 10.1038/s41467-021-23774-w PMC8187432

[43] Zhang W, et al. Graph neural networks and their current applications in bioinformatics. Front Genet. 2021;12:690049. 10.3389/fgene.2021.690049. 34394185 10.3389/fgene.2021.690049 PMC8360394

[44] Entrican G, Charlier J, Dalton L, Messori S, Sharma S, Taylor R, Morrow A. Construction of generic roadmaps for the strategic coordination of global research into infectious diseases of animals and zoonoses. Transbound Emerg Dis. 2021;68(3):1513–20. 32896967 10.1111/tbed.13821

[45] Global Foot-and-Mouth Disease Research Alliance (GFRA). Gap Analysis Report. 2022: 225. https://www.ars.usda.gov/gfra/reports/2022%20Gap%20Analysis%20Workshop%20Report.pdf

[46] STAR IDAZ IRC working group on foot-and-mouth disease. 2024a. https://www.star-idaz.net/priority-topic/foot-and-mouth-disease/

[47] Skuce R., Allen A., Ford T., Romero B., O Brien D., Gormley E., McCormack J., Sawyer J., Bezos J., O Hagan M., Miller M., Coffey M., Gordon S. and Olea Popelka F. DISCONTOOLS chapter on bovine tuberculosis.. Discontools. Discontools. 2024. (Online article)

[48] STAR IDAZ IRC working group on bovine tuberculosis. 2024b. https://www.star-idaz.net/priority-topic/bovine-tuberculosis-btb/.

[49] STAR IDAZ IRC working group on helminths. 2019. https://www.star-idaz.net/priority-topic/helminths-including-anthelmintic-resistance/.

[50] Lim RM, Arme TM, Arinaitwe M, et al. How useful is nanopore adaptive sampling for sequencing *Schistosoma mansoni* miracidia? [version 1; peer review: 3 approved]. Wellcome Open Res. 2025;10:239. 10.12688/wellcomeopenres.24094.1. 40495926 10.12688/wellcomeopenres.24094.1 PMC12149812

[51] Verhoeven JTP, Malwe AS, Roussel N, Nielsen IB, Mak SST, Nielsen TK, Barnes CJ. Adaptive sampling with Oxford Nanopore offers a simple way to improve the efficiency of plant metagenomic studies. New Phytol. 2025;248(4):1620–1624. 10.1111/nph.70450. Epub 2025 Aug 5. PMID: 41097911. 10.1111/nph.70450 PMC12529027 41097911

[52] Moar P, Premeaux TA, Atkins A, Ndhlovu LC. The latent HIV reservoir: current advances in genetic sequencing approaches. mBio. 2023;14(5):e0134423. 10.1128/mbio.01344-23. Epub 2023 Oct 9. PMID: 37811964. 37811964 10.1128/mbio.01344-23 PMC10653892

[53] Olech M, Hodor D, Toma C, Negoescu A, Taulescu M. First molecular characterization of small ruminant lentiviruses detected in Romania. Anim (Basel). 2023;13(23):3718. 10.3390/ani13233718. 10.3390/ani13233718 PMC10705781 38067069

[54] Doyle SR, Laing R, Bartley D, Morrison A, Holroyd N, Maitland K, Antonopoulos A, Chaudhry U, Flis I, Howell S, McIntyre J, Gilleard JS, Tait A, Mable B, Kaplan R, Sargison N, Britton C, Berriman M, Devaney E, Cotton JA. Genomic landscape of drug response reveals mediators of anthelmintic resistance. Cell Rep. 2022;41(3):111522. 36261007 10.1016/j.celrep.2022.111522 PMC9597552

[55] Beesley NJ, Cwiklinski K, Allen K, Hoyle RC, Spithill TW, et al. A major locus confers triclabendazole resistance in fasciola hepatica and shows dominant inheritance. PLoS Pathog. 2023;19(1):e1011081. 10.1371/journal.ppat.1011081. 36701396 10.1371/journal.ppat.1011081 PMC9904461

